# Facilitation of Fast Backward Priming After Left Cerebellar Continuous Theta-Burst Stimulation

**DOI:** 10.1007/s12311-017-0881-6

**Published:** 2017-09-05

**Authors:** Louise S. T. Allen-Walker, R. Martyn Bracewell, Guillaume Thierry, Paloma Mari-Beffa

**Affiliations:** 1 School of Psychology, Bangor University, Bangor, LL57 2AS UK; 2 School of Medical Sciences, Bangor University, Bangor, LL57 2AS UK

**Keywords:** Cerebellum, TMS, Associative priming, Prediction, Backward priming

## Abstract

Traditional theories of backward priming account only for the priming effects found at long stimulus onset asynchronies (SOAs). Here, we suggest that the presence of backward priming at short SOAs may be related to the integrative role of the cerebellum. Previous research has shown that the right cerebellum is involved in forward associative priming. Functional magnetic resonance imaging reveals some activation of the left cerebellar hemisphere during backward priming; but what this activation represents is unclear. Here we explore this issue using continuous theta-burst transcranial magnetic stimulation (cTBS) and associative priming in a lexical decision task. We tested the hypothesis that the left cerebellum plays a role in backward priming and that this is dissociated from the role of the right cerebellum in forward priming. Before and after cTBS was applied to their left and right cerebellar hemispheres, participants completed a lexical decision task. Although we did not replicate the forward priming effect reported in the literature, we did find a significant increase in backward priming after left relative to right cerebellar cTBS. We consider how theories of cerebellar function in the motor domain can be extended to language and cognitive models of backward priming.

## Introduction

In cognitive neuroscience, associative priming is often used to understand how the brain encodes two events taking place in a sequence. In the case of language, some words tend to appear in a particular order, such as DOG-BONE, while they are less frequent in the reversed one (BONE-DOG). When participants are presented with the first word of the pair, responses to the second are usually facilitated, producing a priming effect referred to as “associative” [1]. It is commonly understood that words are represented through associative networks and that the presentation of the prime word (DOG) automatically spreads its activation to those units most closely linked to it (e.g. BONE). When the second word appears, overall responses are facilitated due to its higher level of activation compared to unrelated ones (e.g. ORANGE). This spread of activation is automatic in nature and is considered *not* to be based on expectancies [2], explaining why this effect appears even when the words are presented with a very short interval between them, from a 50- to a 360-ms stimulus onset asynchrony (SOA) [3, 4]. At long SOAs (> 500 ms), priming effects are more commonly attributed to strategic, top–down activation of expected words in memory [1].

A rather puzzling priming phenomenon is what has been termed backward priming, i.e. the improved performance observed when the associated words are presented in the reversed order. Traditional theories of backward priming explain this as a process involving memory retrieval, particularly in lexical decision tasks (LDT). Here participants need to decide whether the second word of a pair is a word or not (word–non-word decision). When the target appears (e.g. DOG), the decision can be helped by strategically retrieving the previous word from episodic memory (e.g. BONE). If they are related, then the target must be a word; but if they are not related, then it could be either a word or a non-word, a conflict that will increase reaction times to unrelated pairs. Being a strategic process, such post-lexical semantic integration takes time and can only account for backward priming with long SOAs [1]. However, backward priming has also been repeatedly observed with short SOAs [5–7], which is inconsistent with the semantic integration theory. Some researchers have suggested that backward priming at short SOAs is due to the same process of spreading activation described above [8]. As Koriat [8] acknowledged, one difficulty posed by these models is that activation normally spreads only in the forward direction, presuming that the prime needs to appear before its target. If we assume that feedback loops connecting prime and target representations do exist, then spreading activation could account for backward priming at short SOA. Here we explore the possibility that these feedback loops might be represented in the cerebro-cerebellar circuits, as part of their wider role as a temporal prediction modeller.

The cerebellum has a very important role in the creation of associations between events or representations that are in a temporal sequence [9], creating both forward and backward links between them to improve both fluency and accuracy. Historically, this function of the cerebellum has been widely studied in sensorimotor control by pairing motor actions to their expected sensorial outcomes and vice versa (see Miall, Weir, Wolpert and Stein [10] for a review of classic models and empirical evidence). However, recent studies show that the cerebellum is also involved in the creation of more abstract relations, such as those involved in verbal working memory [11–14], grammar processing [15, 16] or writing [17] (see Mariën and Manto [18] for a recent review of language functions in the cerebellum). Such a wide range of functions suggest that the cerebellum acts whenever the system needs to link two computational units into a sequence, extending its influence beyond motor control to potentially any representation, including those used in language processing. This is confirmed by a substantial body of research indicating that cerebellar patients have deficits in associative learning across multiple domains including motor control, emotion and cognition (see, e.g. [19, 20] for reviews). Importantly for our purpose, lexical access could be part of these representations, providing a substrate where forward and backward connections can automatically activate each other, and potentially explain backward priming at short SOAs.

When studying the role of the cerebellum in language processing, researchers have employed various techniques, for example by assessing different language functions including lexical and morphological access in cerebellar patients [21, 22], using neuroimaging techniques to reveal the activation of the cerebellum elicited by language-based tasks (e.g. functional Magnetic Resonance Imaging, fMRI techniques [23, 24] and Positron Emission Tomography, PET [25]), functional connectivity [26–28] and neurostimulation techniques such as transcranial direct current stimulation (tDCS; see Argyropoulos [29] for a review), which has recently been combined with fMRI to examine the role of the cerebellum in semantic prediction and how this affects activation in the cerebrum [30]. In particular, researchers have used transcranial magnetic stimulation (TMS) to investigate the role of the cerebellum in predictive or associative priming (for a review, see Beaton, Allen-Walker and Bracewell [31]). Some authors suggest that single-pulse TMS activates the inhibitory Purkinje cells, leading to inhibition of the disynaptic dentato-thalamo-cortical facilitatory connections, which, in turn, leads to inhibition of the primary motor areas and prefrontal cortex in the contralateral cerebral hemisphere [32–34]. Conversely, other investigators have suggested that behavioural facilitation in motor and non-motor domains involves cerebellar suppression, rather than activation (e.g. [35, 36]), perhaps by suppressing the inhibitory Purkinje cells. Indeed, within the context of cerebellar TMS and language association, both inhibitory and facilitatory behavioural effects have been reported; specifically, repetitive transcranial magnetic stimulation (rTMS) effects have been inhibitory [37], whereas cTBS effects have tended to be facilitatory [38, 39].

TMS studies have not explored the role of the cerebellum in backward priming but have instead focused on forward priming. For example, Argyropoulos [38] used continuous theta-burst stimulation (cTBS) to test both phrasal associative priming (e.g. gift ➔ HORSE) and a type of categorical priming where the prime was a subordinate of the target (e.g. apple ➔ FRUIT) in a lexical decision task. The author compared medial (1 cm below and 1 cm to the right of the inion) and lateral (1 cm below and 4.5 cm to the right of the inion) stimulation of the right cerebellum. cTBS over the medial site selectively enhanced phrasal associative priming as compared to categorical priming, demonstrating a role of the right cerebellum in forward priming. A subsequent study [39] also found increases in noun-to-verb associative priming (scissors ➔ cutting) after stimulating other areas of the right cerebellum, although this time they were located in more distant lateral sites (1 cm below, 10 cm lateral of inion). In this case, the direction of the associative relation was not described and they used a different list of associated pairs, potentially explaining the difference in relevant locations. In any case, these two studies provide evidence for a role of the right cerebellum in associative and forward priming, opening the possibility that it could also be involved in associative backward priming. Nevertheless, other studies using right vermal stimulation from the same laboratory failed to replicate this finding [40], clouding the role of the right cerebellum in creating associations between word pairs.

Argyropoulos [38] posits that the role of the cerebellum in language is predictive, placing this role in the context of widely held theories of the predictive role of the cerebellum in motor control [41–43]. The cerebellum is homogenous in its internal architecture [44], leading to speculations that it performs a similar computation in non-motor and motor domains, such as that of verbal working memory [11], and other types of higher order cognitive processes [45, 46], in our case language [47].

Further support for the predictive model of motor function applied to language comes from Lesage et al. [37]. The authors applied 1 Hz offline rTMS to the right cerebellar hemisphere (1 cm below and 3 cm lateral of the inion) with the vertex as a control site. Participants listened to sentences in which the final noun could be predicted on the basis of the verb (e.g. “The man will sail the boat”) or not (“The man will watch the boat”). Simultaneously, four different pictures were displayed on the screen and participants had to fixate on the picture depicting the final noun. Thus, the latency of the eye movement could be taken as an index of the predictability of the noun. They found that eye movement latencies for the predictable sentences were affected after stimulation of the right cerebellum but not the vertex, thus favouring the interpretation of a predictive role of the cerebellum in the representation of unidirectional associations. Using a similar protocol, Miall and colleagues [40] replicated this finding with tDCS. They found that, for sentences with verbs specifically indicating a final noun, anodal tDCS over the right cerebellum reduced response latencies when predicting the final word, whereas cathodal increased response latencies. This effect was not present for the sentences with low predictability where general verbs did not indicate the final noun, further supporting the role for the cerebellum in language prediction.

Although no TMS studies have yet been conducted to establish whether the cerebellum is involved in backward priming, there is some indirect evidence in the fMRI study by Terrien et al. [7]. They examined forward and backward priming with short SOAs using fMRI to determine their neural correlates. They found activation of the right cerebellum in forward and backward priming, and of the left cerebellum in backward priming, combined with activation in the right middle temporal gyrus. This result suggests that forward and backward priming might be supported by separate functional brain networks. Furthermore, the asymmetrical distribution of these networks across the hemispheres makes them good candidates for cerebellar cTBS.

In the current study, we examined the role of the cerebellum in both backward and forward priming with a short SOA during a lexical decision task. Based on results obtained in previous studies [37–39], we expected modulation of predictive processing as indexed by changes in forward priming following right cerebellar cTBS. In addition, following Terrien et al. [7], we anticipated modulation of backward priming following left cerebellar cTBS.

## Methods

### Participants

Sample size was estimated a priori using G*Power 3.1 [48]. For a desired power of 0.90 or above, an expected effect size of 0.25 or above and an alpha of 0.05, we estimated the required sample size for this 2 × 2 × 2 analysis of variance (ANOVA). The minimum repeated measures correlation that we ever observed in this task across any pair of conditions was 0.6 producing a minimum required sample of 16 participants.

Nineteen students from Bangor University participated (nine males, between the ages of 20 and 30 years, *M* = 24.2, SD = 2.1). Due to overall poor performance on the task (overall reaction times—RTs—falling three box lengths above the median in a box plot), the data from a twentieth participant were discarded. The 18 right-handed participants and the left-handed participant were all native speakers of English, with normal or corrected-to-normal vision. The pattern of results for the left-handed participant did not differ from that of the right-handed ones. Standard exclusion criteria for TMS studies were applied: Participants were not selected if they had an artificial heart valve, ever had metal fragments in their eyes, ever had any metal or shrapnel in their body, ever had any implanted electrical devices, had any heart problems, had participated in a brain stimulation experiment within the last 7 days or if they had been stimulated before with adverse effects, if they had ever suffered from a neurological or psychiatric illness, if anyone in their family had a history of seizures, had a history of fainting, suffer from migraines, had recently been binge drinking or taken recreational drugs or if they were pregnant. The participants were tested following the safety guidelines established by Bangor University. The procedure and experiment were approved by the Ethics Committee of the School of Psychology at Bangor University, and every participant gave their informed consent before taking part.

### Stimuli

Participants were presented with 144 related word pairs: 24 pairs of forward associatively related words (e.g. cardboard ➔ BOX), 24 pairs of backward related words (e.g. box ➔ CARDBOARD) and 48 pairs of associatively unrelated filler words (e.g. knife ➔ UTENSIL), all presented twice. Associative pairs were of two types: 12 asymmetrically associated pairs and 12 compound words following common practice in the backward priming literature [5, 49, 50]. We used the University of South Florida Word Association Norms [51] to select the asymmetrically associated pairs. From these, we chose those with the highest level of recognition in British English after piloting them with a sample of our postgraduate students. As a result, the associative strength was significantly higher for the forward pairs (0.1%) than for the backward pairs (0.008%; *t*(11) = 2.56, *p* = .026) with no overlap in associative strengths between the two directions. Unrelated pairs had zero associative strength in all cases, which was significantly different from the forward associative pairs (*t*(22) = 2.31; *p* = .031), but not different from the backward ones (*t*(22) = 1; *p* = .329). Associative (forward/backward) and unrelated pairs did not differ in terms of semantic similarity [52] (path length of 0.152 and 0.147 respectively, *p* = .864), demonstrating that potential differences should be free of categorical semantic confounds.

Participants were also presented with 48 unrelated word pairs. From these, 24 were constructed to match the forward associatively related pairs using the same primes and reassigning them to new targets with zero associative relatedness (e.g. cardboard ➔ BOY). The same was done with 24 unrelated pairs designed to match the primes of the backward associatively related pairs (e.g. box ➔ CROSS). Each pair type was presented twice in the course of the experiment, resulting in 96 associatively unrelated pairs overall, used to measure priming.

Participants were also presented with 288 non-word pairs, constructed using the same primes as described above and with target words changed to non-words and re-associated with different primes (e.g. cardboard ➔ DUWN). The non-word targets were pseudowords created by either changing a vowel to another vowel or swapping two consonants, ensuring that all resulting stimuli were pronounceable but had no known meaning in either English or Welsh. As a result, each prime word was presented paired with three types of target (related, unrelated, pseudoword) within each testing session. Half of the targets were real words and the other half were non-words (see Table 1).

**Table 1:** Example stimuli

Stimulus type	Related	Unrelated	Non-word
Forwards	Pigeon ➔ HOLE	Pigeon ➔ BACK	Pigeon ➔ BOCK
Backward	Hole ➔ PIGEON	Hole ➔ BOOK	Hole ➔ BOEK

Lexical frequency was obtained for primes and targets from the CELEX lexical database [53] using the N-Watch program [54]. There was no significant difference in lexical frequency between the primes and the targets, *t*(23) = − 0.54, *p* = .59. There was also no significant difference in length between primes and targets, *t*(23) = 1.64, *p* = .115 (Table 2).

**Table 2:** Means and standard deviations for frequency and length

Stimuli type	Prime frequency	Target frequency	Prime length	Target length
Forwards	164.88 (257.69)	218.70 (394.89)	4.75 (1.57)	4.08 (1.06)
Backward	218.70 (394.89)	164.88 (257.69)	4.08 (1.06)	4.75 (1.57)

Thus, overall, participants were presented with 576 trials, 288 featuring word targets (“Yes” responses) and 288 featuring non-word targets (“No” responses). From the Yes responses, 96 corresponded to associatively related targets (48 forward related and 48 backward related) and 192 to associatively unrelated ones (48 used as control for the forward pairs, 48 used as control for the backward pairs and 96 fillers). Associative relatedness proportion was 0.2, which is low enough to prevent participants from engaging in top–down strategies [1, 4]. The same stimuli were presented in each testing session, and the trial order was random for each participant and each phase.

### Task

Participants were asked to respond as quickly and accurately as possible via button press in a lexical decision task (LDT). Keys “M” and “Z” on a standard QWERTY keyboard were used, one for existing words and the other for non-words, and response sides were counterbalanced by cTBS hemisphere and between participants, i.e. for both pre- and post-right cTBS, M corresponded to real words and for pre- and post-left cTBS, Z referred to a real word, or vice versa. In each trial, a fixation cross was presented for 250 ms, then the prime for 150 ms, then the target was presented until response. After the response had been made, there was a 500 ms interval before the next trial began.

### TMS Apparatus

Stimulation was delivered using a 70-mm figure of eight shaped coil connected to a Magstim Super Rapid Transcranial Magnetic Stimulator (Magstim, Whitland, UK). The coil was positioned tangentially to the scalp with the handle pointing upwards, producing a downward current in the cerebellum. This coil position has proven optimal for suppressing the contralateral motor cortex in single-pulse TMS (e.g. [55]) and has been shown to successfully interfere with cognitive processes such as procedural learning in 1-Hz rTMS paradigms (e.g. [56]).

### TMS Locations

cTBS was applied to the left and right cerebellum, 1 cm below and 3 cm lateral to the inion. This is likely to stimulate posterolateral regions of lobules HVI/HVIIa Crus I/II [57, 58], but see Argyropoulos [58] for a discussion about the difficulties to accurately identify the stimulated areas when using this type of coil. This location has previously been shown to be an effective area to stimulate when trying to affect the right cerebellar hemisphere’s predictive function [37, 59].

### TMS Protocol

A cTBS protocol was used. A burst of three pulses was delivered at 50 Hz frequency; this burst was repeated at an interval of 200 ms; the whole run lasted for 40 s (given 600 pulses in total). This protocol has previously proven reliable for producing behavioural change [38, 39, 60] and has also been shown to be well tolerated and safe [61].

### TMS Intensity

The stimulation intensity was set at 55% of maximum stimulator output (MSO) for all participants. Although TMS experimenters often define their stimulation intensity on the basis of each participant’s motor threshold, several recent cerebellar TMS studies have used fixed intensities as this procedure is more appropriate for cerebellar stimulation [37–39].

### Procedure

To begin with, participants were fully informed of the risks associated with TMS. They were given a brief explanation of the history of the method and how it acts on the brain. After screening and informed consent, participants were given the opportunity to experience the sensation of TMS. Single pulses were delivered at the approximate site of stimulation beginning at 30% of MSO and rising in increments of 5 to 55% of MSO. These single pulses were only delivered during the first session. At any point, the participants could choose to stop and withdraw if they found the sensation too uncomfortable. Following an interval of 15 min, the first pre-stimulation session of the LDT was then completed, followed by the stimulation. After a 7-min delay participants performed the LDT again, since a delay after the administration of cTBS has been shown to enhance behavioural effects [62]. Throughout the session, the participants were asked to stay seated in the same chair to avoid disruption of the effect of the cTBS on the cerebellum. After a week, the participants returned and completed a second session structured in the same way as the first session but without the information and consent, which was designed to cover both sessions.

### Design and Analyses

The order of stimulation sites was fully counterbalanced across participants, and order effects were compared between groups (right–left, left–right) before any other analyses were conducted.

We compared RTs before and after each cTBS session (pre–post—from now on referred to as phase). In addition, we compared the side of stimulation, left or right cerebellar hemispheres. Finally, priming effects for forward and backward pairs were calculated by comparing related and unrelated conditions,^1^ as described in the “Stimuli” section. All the RT analyses were replicated with accuracy data except those of priming sizes.

RT data were extracted by first eliminating responses to the first 10 practice trials and then averaging all correct Yes responses with RTs less than 2 SD away from the mean for each participant and in each condition. This data filtering was applied separately to forward and backward conditions. The resulting means were then submitted to a 2 phase (pre, post) × 2 hemisphere (left, right) × 2 relatedness (related, unrelated) repeated measures ANOVA. This was followed up by the analysis of priming sizes using a 2 phase (pre, post) × 2 hemisphere (left, right) design, again for both forward and backward pairs. Priming sizes were calculated according to convention [1], RT for unrelated stimuli minus RT for related ones.

Accuracy data were calculated after filtering and represent the proportion of correct Yes answers excluding the first 10 practice trials, and they were analysed using the 2 × 2 × 2 design described above.

## Results

Results (both RT and accuracy) from participants who received cTBS on the right first were compared to those who received the stimulation in the reverse order. There was no main effect of order nor any interaction with the other variables in the design. Therefore, data were collapsed across groups for further analysis. RT and accuracy data per participant per condition were analysed using a 2 (phase) × 2 (hemisphere) × 2 (relatedness) repeated measures ANOVA separately for backward and forward trials.

### Backward Priming

Analysis of RTs for backward pairs showed that participants were overall 20 ms faster after TMS than before [*F*(1, 18) = 34.72, *p* < .001, *η*_*p*_^2^ = 0.66]. No overall effect of hemisphere was found (*F* < 1). Participants were also 13 ms faster on average in related as compared to unrelated trials [*F*(1, 18) = 58.09, *p* < .001, *η*_*p*_^2^ = 0.76]. Importantly, there was a significant interaction between phase, hemisphere and relatedness [*F*(1, 18) = 4.05, *p* = .05, *η*_*p*_^2^ = 0.18]. No other interactions were significant.

**Fig. 1:**
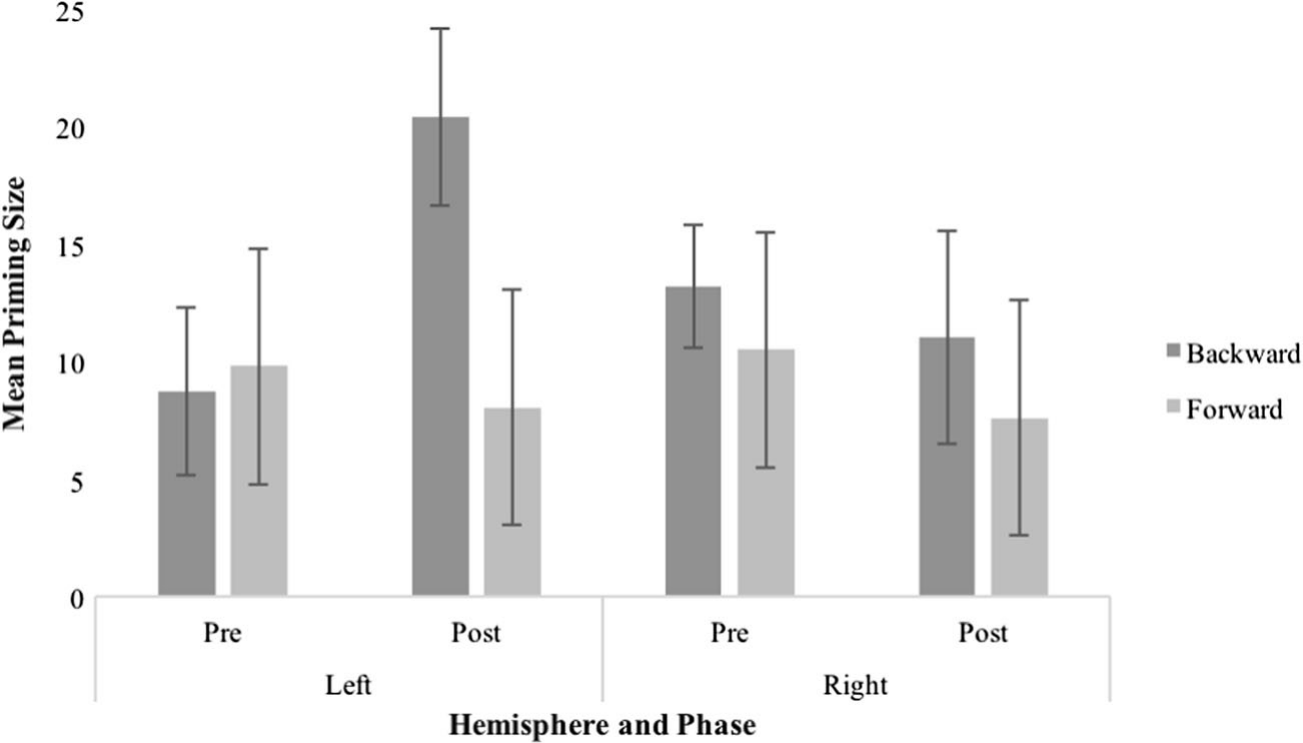
Mean priming size (unrelated reaction time minus related reaction time in ms) for backward and forward word pairs, split by phase and hemisphere. Error bars depict ± 1 standard error of the mean

To further investigate the three-way interaction, we analysed changes in backward priming size across phase (pre–post) and hemisphere (left–right). Backward priming was increased exclusively after left hemisphere stimulation [15 ms larger, *t*(19) = 3.44, *p* = .003], retaining virtually the same size when the right hemisphere was involved (− 2 ms; Fig. 1).

There was no significant effect of any variable on accuracy rates (Table 3).

**Table 3:** Mean RTs (in ms), standard deviations, and average accuracy percentages per condition

			Pre-left	Pre-right	Post-left	Post-right
Backward	Related	M (Sd) %	519 (63) 95	516 (60) 96	493 (60) 94	498 (71) 95
	Unrelated	M (Sd) %	527 (67) 93	529 (61) 94	513 (62) 92	509 (60) 93
Forward	Related	M (Sd) %	523 (59) 95	526 (67) 94	500 (63) 94	498 (56) 94
	Unrelated	M (Sd) %	533 (58) 91	537 (62) 92	508 (57) 91	505 (50) 92

*M* arithmetic mean, *Sd* standard deviation, *%* percentage of correct responses

### Forward Priming

As with the backward pairs, overall RTs were 28 ms faster overall after cTBS than before [*F*(1, 18) = 59.14, *p* < .001, *η*_*p*_^2^ = 0.77]. No effect of hemisphere was found (*F* < 1). Related targets were responded to 9 ms faster than unrelated ones [*F*(1, 18) = 8.91, *p* = .008, *η*_*p*_^2^ = 0.33]. No interactions were significant.

Responses to related targets were 3% more accurate than unrelated ones [*F*(1, 18) = 6.40, *p* = .02, *η*_*p*_^2^ = 0.26]. There was no other significant effect of any variable on accuracy.

All statistical outcomes for the above analyses are displayed in Table 4.

**Table 4:** Table of statistical outcomes (*p* values and effect sizes) for all ANOVAs conducted for mean RTs, priming size and accuracy

		Backward		Forward	
RT		*p* value	${\eta}_p^2$	*p* value	${\eta}_p^2$
Main effects	Phase	.00***	0.66	.00***	0.77
	Hemisphere	.97	0.00	.97	0.00
	Relatedness	.00***	0.76	.01**	0.33
Interactions	Phase × hemisphere	.85	0.00	.38	0.04
	Phase × relatedness	.11	0.13	.54	0.02
	Hemisphere × relatedness	.61	0.02	.97	0.00
	3-way interaction	.05*	0.18	.88	0.00
Priming size					
Main effects	Phase	.11	0.13	.54	0.02
	Hemisphere	.61	0.02	.97	0.00
Interactions	Phase × hemisphere	.05*	0.18	.88	0.00
Accuracy					
Main effects	Phase	.07	0.17	.31	0.06
	Hemisphere	.06	0.19	.53	0.02
	Relatedness	.06	0.19	.02*	0.26
Interactions	Phase × hemisphere	.96	0.00	.86	0.00
	Phase × relatedness	.81	0.00	.80	0.00
	Hemisphere × relatedness	.93	0.00	.47	0.03
	3-way interaction	.90	0.00	.48	0.03

*p ≤ .05; **p ≤ .01; ***p < .001

### Ratios Analysis

Further analyses were conducted using related/unrelated ratios as a measure of priming. These produced identical results with no changes across the hemispheres due to stimulation found with forward priming (*F* < 1) and a strong increase in priming following left hemisphere stimulation in the case of backward priming (*p* = .026).

## Discussion

In this study, we investigated the role of the cerebellum in backward associative priming at short SOAs in a lexical decision task. To summarise our argument, backward priming is commonly explained as a result of strategic processes of episodic post-lexical integration [1] that are understood to take time and therefore require long SOAs. Backward priming at short SOAs represents a challenge for this theoretical account [49] and has been attributed to fast and automatic spread of activation in associative networks [2, 8]. Such networks are thought to be built on unidirectional connections, where the prime needs to appear before the target for priming to occur [8]. Backward priming would thus need the inclusion of feedback loops in the associative network [8]. Some previous studies have pointed to the cerebellum as a likely locus for the representation of these associations both in forward [38, 39] and backward priming [48]. Therefore, we examined the impact of right and left cerebellar cTBS on forward and backward associative priming with short SOAs.

Here, we used an interval of 150 ms between prime and target onsets, making it unlikely that top–down mechanisms would account for a backward priming effect. Participants were generally faster after cTBS regardless of the stimulated hemisphere, probably due to practice effects. However, a significant increase in the priming effect was found only for backward related stimuli after left hemisphere stimulation, in the absence of any change for forward priming. This validates our hypothesis that backward priming at short SOAs critically involves the left cerebellar hemisphere. In addition, we have found some preliminary evidence that feedback loops in the associative network can be dissociated from forward connections and that they could critically involve the left cerebellar hemisphere.

A role of the left cerebellar hemisphere in backward priming is consistent with previous fMRI research. Terrien et al. [7] found activation for backward priming in the right inferior occipital gyrus and the middle temporal gyrus at short SOAs. It has been proposed that these areas were interpreted as being responsible for mechanisms of post-lexical integration. In the Terrien study [7], the authors found activation in the left cerebellum, but they did not hypothesise a role for this region in priming.

Indeed, the presence of automatic and fast feedback loops in the left cerebellar hemisphere may explain why Terrien et al. [7] found activation in the left cerebellum during backward priming at short SOAs. Given that the left cerebellum interacts preferentially with the contralateral cerebral hemisphere [63], it could feed into a wider lexical processing system, possibly involving the right occipitotemporal network [64]. This would help explain why some authors have found right cerebral activation during backward priming [24, 65]. In any case, no activation in the left cerebellum was found in studies using long SOAs, suggesting that it has a more specific role in the formation of automatic associations rather than episodic ones.

Further evidence of this cerebellocerebral network has been reported by Cho et al. [66]. In a large-scale functional connectivity study, Cho and colleagues found metabolic changes in the contralateral right temporal cortex as a result of left cerebellar rTMS, stimulating a region similar to the one targeted here (1 cm below and 3 cm lateral of the inion), using 18F-fluorodeoxyglucose (FDG) PET. Interestingly, this activation also spread to different bilateral cortical areas typically involved in language (such as Broca’s and Wernicke’s areas), suggesting an even wider implication of this functional network in higher order cognitions, specifically language.

Feedback loops in the cerebellum have long been considered an explanation in the formation of automatic, predictive, sensorimotor associations and thus to be responsible for the fluency and accuracy of sensory-guided actions [10]. Moberget et al. [47] used fMRI to show that these models of cerebellar motor function are transferable to language. The authors presented sentences in which the final target was highly predictable, such that congruent sentences featured an expected word, whereas the incongruent sentences ended in an unexpected fashion. They found activation in the right cerebellar hemisphere when the target word was predictable and a higher level of activation when the prediction was violated. They proposed that this pattern of activation is consistent with models of sensorimotor control, supporting the idea that cerebellar computation may extend to the domain of abstract associations (including that of verbal working memory [11]). These studies, however, have focused mostly on the forward aspect of prediction within such models. Our study contributes to this area by showing that these models also apply to feedback loops in language processing. A good example of feedback loop involvement in sentence comprehension is when a particular word requires contextual disambiguation from words presented later in the sentence. For instance, homophones can sometimes be differentiated only after presentation of disambiguating contextual information (e.g. the bank ran out of money/the bank was flooded). The appearance of the disambiguating context re-activates the prime word with an updated meaning [8]. In addition, some theories combine spreading of activation models with feedback in the context of sentence production, as in the case of correction during slips of the tongue errors [67].

Our experiment was designed to test backward priming and thus substantially differs from the original studies by Argyropoulos and colleagues [38, 39]. So, it is not surprising that we did not fully replicate their results regarding forward priming at short SOA following right cerebellar cTBS. The inclusion of backward priming led us to include word pairs that are strongly asymmetric, including a mixture of compound and non-compound words unlike those used by Argyropoulos and colleagues. Considering existing cerebellar stimulation evidence, there seems to be a trend towards right-sided effects with predominantly verb to noun priming. It is not yet clear whether this trend also holds for other types of associative pairs. For example Argyropoulos, Kimiskidis and Papagiannopoulos [40] did not replicate the results obtained by Argropoulos [38] with noun to noun associative pairs that were not otherwise related (e.g. gift ➔ HORSE). Another difference between our study and previous ones is that, unlike Argyropoulos and colleagues, we used the same set of stimuli across all sessions and conditions. While this choice optimises the consistency and comparability across conditions, it increases the likelihood of practice effects arising and perhaps explains the effect of phase in overall RTs. It is noteworthy that Argyropoulos and colleagues [68] found disruption of practice effects due to vermal cerebellar stimulation. In contrast, evidence from tDCS studies indicate that the use of cerebellar stimulation at multiple time points does not modulate learning effects arising from the repeated presentation of the same stimuli [40], where the sites used (1 cm below the inion and 2 cm to the right) are proximal to those used in the present study. In any case, we do not expect that our choice to repeat stimuli is responsible for the increased backward priming effect after left, compared to right stimulation, as the practice is identical in both.

One of the most important differences to note across studies is the difference in stimulation sites (3 cm laterally in the present study; 1 cm laterally in Agryropoulos [38]; 10 cm laterally in Argyropoulos and Muggleton [39]). At this point, it is important to highlight that the same authors have also reported a lack of priming modulation after cerebellar stimulation at different sites, such as 4.5 cm lateral of the inion [38, 68]. Therefore, it is possible that forward priming effects involve areas that were unaffected by the stimulation in our experiment. With respect to backwards priming, there is no previous TMS study conducted that we could have used to guide our choice of stimulation site. The only anatomical reference that we found appeared in the Terrien et al. paper [7]. However, the location coordinates reported an area deep into the cerebellum difficult to stimulate with our coil. Our choice of site was driven by a need to be consistent with other ongoing experiments in our laboratory while replicating the sites used in other studies linking it to predictive function [37, 57]. Such studies mostly used sentences, while we used single word presentation. The fact that we did not find a significant increase in forward priming for the right cerebellar hemisphere with these sites may be due to differences in the stimuli and tasks used. Further replication of this study using the sites targeted by Argyropoulos and colleagues might demonstrate a specialization not only of hemispheres but also of areas within them, for forward and backward priming, respectively.

A further possible limitation of this experiment is that we did not employ MRI-guided cTBS, which has previously been discussed as a general issue in this context [58]. MRI-guided cTBS been previously used in cerebellar research domains such as working memory [61] and language processing [60]. With this technique, there is a much larger chance of accuracy in terms of stimulating the areas of interest, providing a better guarantee of modulation within the intended area. Additionally, the use of fMRI and MRI-guided TMS normally results in larger effect sizes, requiring fewer participants to produce significant effects [40]. Although it does not invalidate the results reported here and elsewhere [38, 39], the use of MRI-guided cTBS is likely to increase the resolution of our findings in the future.

Another possible limitation of our study related to the use of a figure-of-eight shaped coil, which has been shown to be less effective that other coils (batwing and double-cone) when stimulating cerebellar sites, particularly at a low intensity [19]. It must be noted, however, that the impact of coil type on cerebellar stimulation has predominantly been studied in the domain of motor function and it is unclear whether this can be readily applied to the domain of cognition. In fact, the figure-of-eight shaped coil remains the most commonly used coil in cerebellar studies of language processing [e.g., 20, 38, 39].

Future research could employ a combination of cTBS and fMRI, perhaps using a similar protocol as that used here (pre- and post-cTBS), during an associative priming task. Previous research has shown that rTMS over the cerebellum has effects on language-related regions of the cortex [66]. Even though the combination of fMRI and cTBS has not been used to examine the relationship between language prediction and later functional changes at the cortical level, fMRI research has revealed activations in Broca’s area and Wernicke’s area in addition to the cerebellum during semantic violation tasks [47]. That being said, tDCS has been recently used in combination with fMRI during a semantic prediction task [40] to more specifically characterise brain areas (e.g. right cerebellar Crus I/II) linked to semantic predictive function. By combining cTBS and fMRI in the future, we may be able to account for some of the disparities in the location of stimulation reported previously.

To conclude, we report evidence that the left hemisphere of the cerebellum is involved in backward associative priming at short SOAs. cTBS applied to the left cerebellum specifically reduced RTs to related, relative to unrelated, stimuli inducing enhanced priming. It is therefore likely that forward and backward priming critically involve different areas of the cerebellum. These results are important for current theories of backward priming, especially at short SOAs, since they point to a potential contribution of cerebellar feedback loops in predictive associative networks. It also extends the involvement of the cerebellum in predictive association beyond sensorimotor control to the sphere of cognitive functioning.

## Footnotes

1 The related but associatively unrelated pairs were intended to be used as fillers. We nevertheless did some exploratory analyses and confirmed that they behaved similarly to those in the unrelated condition. Priming effects were also observed when compared to forward and backward pairs, and no interaction reached significance in this analysis. Because the fillers were not matched in any aspect to the associative pairs, we have no basis for interpreting the outcomes.
